# Nanoparticles in the battle against *Candida auris* biofilms: current advances and future prospects

**DOI:** 10.1007/s13346-024-01749-w

**Published:** 2024-11-26

**Authors:** Bahgat Fayed

**Affiliations:** Department of Chemistry of Natural and Microbial Products, Pharmaceutical and Drug Industries Research Institute, National Research Centre, 33 El Bohouth Street, P.O. Box 12622, Dokki, Giza, Egypt

**Keywords:** Biofilm, Multidrug resistance, Nanoparticles, Antifungal therapy

## Abstract

*Candida auris* has emerged as a significant global health threat due to its multidrug resistance and ability to form robust biofilms, particularly on medical devices and hospital surfaces. Biofilms protect *C. auris* from antifungal treatments and the host immune response, making infections persistent and difficult to control. This review explores the potential of nanoparticles to overcome the limitations of traditional antifungal therapies in combating *C. auris* biofilms. Nanoparticles, with their unique physicochemical properties, offer promising strategies to penetrate biofilm matrices, deliver antifungal agents, and disrupt biofilm structure. Various types of nanoparticles, including metallic, polymeric, lipid-based, and cyclodextrin-based, demonstrate enhanced biofilm penetration and antifungal activity. Their ability to generate reactive oxygen species, disrupt cell adhesion, and release antifungals in a controlled manner makes them ideal candidates for biofilm-targeted therapies. This review presents the current advancements in nanoparticle-based solutions, emphasizing the need for further research into their mechanisms of action, safety, and clinical application. By addressing the challenge of *C. auris* biofilms specifically, this review provides a critical synthesis of existing knowledge and identifies future directions for developing effective antifungal therapies using nanotechnology.

**Graphical abstract:**

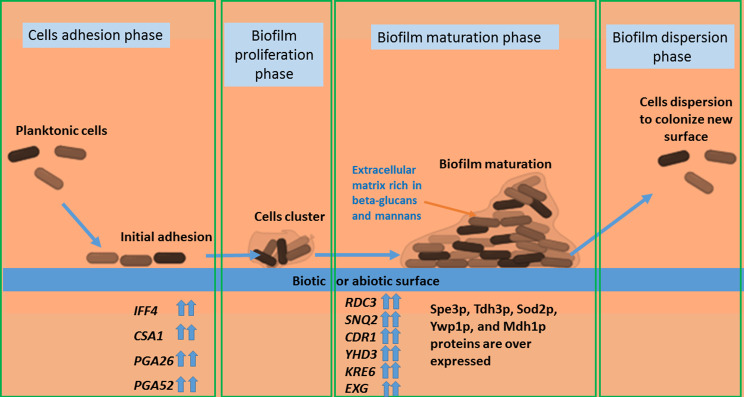

## Introduction

*Candida auris* (*C. auris*) has emerged as a highly problematic multidrug-resistant fungal pathogen, posing significant challenges to global healthcare systems. First identified in 2009, *C. auris* has swiftly spread across multiple continents, causing serious infections in vulnerable populations, particularly those with compromised immune systems or underlying health conditions [1, 2]. Its ability to cause severe invasive infections, coupled with its alarming resistance to multiple classes of antifungal drugs—including azoles, echinocandins, and polyenes—has made *C. auris* a major concern in both clinical and infection control settings [3, 4].

A particularly concerning feature of *C. auris* is its propensity to form robust biofilms on various surfaces, including medical devices such as catheters and ventilators [5]. These biofilms are dense clusters of fungal cells embedded in a protective extracellular matrix, which shield the organisms from both host immune responses and antifungal treatments [6]. The biofilm mode of growth significantly complicates treatment, as traditional antifungal therapies are less effective against biofilm-associated infections compared to planktonic (free-living) forms of the fungus. Consequently, *C. auris* biofilms contribute to its persistence in healthcare environments and its potential for causing outbreaks [7]. Additionally, this biofilm is enriched with unique components, such as mannan-glucan complexes, which are less prominent in other *Candida* biofilms and hinder drug penetration [8]. Compared to *C. albicans* and other fungi, the biofilm of *C. auris* has more active efflux pumps, which expel antifungal agents [9].

In light of these challenges, nanoparticles have emerged as a promising alternative or adjunct to conventional antifungal treatments [10]. Nanoparticles, with their unique physicochemical properties, offer several potential advantages in combating *C. auris* biofilm. These include enhanced delivery of antifungal agents to biofilm-embedded cells, disruption of biofilm matrices, and direct antimicrobial activity against both planktonic and biofilm forms of the fungus [10, 11]. Nanoparticles can be engineered to target specific components of the biofilm or to deliver drugs in a controlled manner, potentially overcoming some of the limitations associated with traditional antifungal therapies [12].

This review aims to provide a comprehensive examination of the current state of research on nanoparticles in the context of *C. auris* biofilm management. It will cover various types of nanoparticles, including metallic, polymeric, and lipid-based nanoparticles, and their mechanisms of action against *C. auris* biofilms. Additionally, the review will discuss recent advancements in nanoparticle-based therapies, and the challenges that need to be addressed for their successful implementation. By synthesizing existing knowledge and highlighting future research directions, this review seeks to advance the understanding of how nanoparticles can be harnessed to address one of the most pressing challenges in fungal infection management today.

## Emergence and global spread

*C. auris* was first identified in 2009 from a patient’s ear canal in Japan, marking the beginning of its recognition as a significant fungal pathogen [1]. Since its discovery, *C. auris* has demonstrated an alarming capacity for global dissemination, becoming a critical concern for healthcare systems worldwide [13]. The emergence of *C. auris* has been characterized by its rapid spread across continents, with documented cases across Africa, Asia, Europe, the Americas, and Oceania [2]. The global spread of *C. auris* is partly attributed to its ability to persist in healthcare environments. The fungus can survive on surfaces and medical equipment for extended periods, often resisting standard cleaning and disinfection protocols. This environmental persistence, combined with the increasing movement of patients between healthcare facilities, contributes to its widespread transmission [14]. The pathogen’s resistance to multiple classes of antifungal drugs exacerbates the situation, complicating treatment efforts and leading to outbreaks in hospitals and long-term care facilities [10].

The spread of *C. auris* has been further facilitated by its ability to form resistant biofilms on medical devices and surfaces, providing a protective environment that contributes to its resistance against both antimicrobial treatments and host immune responses [5, 15]. This biofilm-forming ability not only aids in the persistence of the pathogen but also complicates infection control measures. Moreover, the emergence of different clades of *C. auris*—including South Asian (Clade I), East Asian (Clade II), African (Clade III), South American (Clade IV), and Iranian (Clade V)—reflects the pathogen’s diverse genetic background and varying patterns of drug resistance [16]. Each clade exhibits unique resistance profiles, with some strains demonstrating pan-resistance to all major antifungal classes, further complicating treatment and infection control efforts [2].

## Significance and treatment challenges of *Candida auris* biofilms

The ability of *C. auris* to form biofilms is a central factor contributing to its clinical significance and the difficulty of managing infections caused by this pathogen. Biofilms are complex, structured communities of microbial cells embedded in a self-produced extracellular matrix of polysaccharides, proteins, and nucleic acids. The biofilm formed by *C. auris* exhibits several distinctive characteristics that contribute to the unique challenges in treating *C. auris* biofilm infections.

### Enhanced resistance to antifungal therapy

*C. auris* biofilms are remarkably resistant to antifungal treatments compared to their planktonic (free-floating) counterparts. For example, Romera et al. investigated the minimal inhibitory concentration (MIC) for three strains of *C. auris*, revealing that while the planktonic forms were sensitive to echinocandins and polyenes, the biofilm forms exhibited significant resistance [15]. Similar findings were reported by Sherry et al. [17]. The extracellular matrix of biofilms serves as a physical barrier, impeding the penetration of antifungal agents and diminishing their efficacy [18]. Additionally, *C. auris* biofilms are enriched with a mannan-glucan complex, which can sequester antifungal drugs and prevent them from reaching their intracellular targets [19]. Furthermore, cells within the biofilm can enter a dormant or less metabolically active state, making them less susceptible to drugs that primarily target actively growing cells [20]. Notably, a unique characteristic distinguishing *C. auris* from other *Candida* species is the upregulation of several genes encoding efflux pumps, including ATP-binding cassette (ABC) and major facilitator superfamily (MFS) transporters, during biofilm development, which can contributes to *C. auris* biofilm resistance against antifungal drugs [21].

### Protection from host immune response

Generally, *Candida* biofilm is surrounded by a dense extracellular matrix that serves as a physical barrier, hindering immune cells, such as neutrophils and macrophages, from effectively accessing and engulfing the fungal cells [22]. Of particular interest is the ability of *C. auris* biofilm to resist neutrophil attack, a feature that distinguishes it from its planktonic counterparts [23]. Unlike planktonic cells, which are relatively more susceptible to immune clearance, *C. auris* biofilm exhibits a robust defense mechanism that significantly enhances its survival within the host. This resistance is not solely due to the physical barrier created by the extracellular matrix but also involves complex interactions between the biofilm and the host immune system. A distinctive feature of *C. auris* biofilm is its altered composition of mannans and β-glucans, which play crucial roles in immune evasion. These polysaccharides, which are key components of the fungal cell wall, exhibit lower binding affinity and reduced recognition by pattern recognition receptors (PRRs) on immune cells compared to those found in other *Candida* species [24]. PRRs, such as Dectin-1 and Toll-like receptors, are essential for recognizing fungal pathogens and initiating immune responses [5]. However, the modified mannans and β-glucans in *C. auris* biofilm impair this recognition, thereby diminishing the effectiveness of the immune response.

These characteristics are key aspects of how *C. auris* biofilm protects itself from the host immune system. By evading immune detection and resisting neutrophil attacks, *C. auris* biofilm not only persists within the host but also poses a significant challenge in clinical management, contributing to the pathogen’s high morbidity and mortality rates. Understanding these mechanisms is crucial for developing novel therapeutic strategies aimed at disrupting biofilm formation and enhancing the host’s ability to combat *C. auris* infections.

### Pathogenesis

The pathogenesis of *C. auris* in its biofilm form differs markedly from that of its planktonic cells, contributing to more severe and persistent infections. This difference is primarily driven by the unique molecular and physiological adaptations that *C. auris* undergoes during biofilm formation. Transcriptomic analysis of *C. auris* biofilms has revealed the upregulation of genes crucial for fungal pathogenicity, including *PLB3* and *SAP5* [21]. These genes encode phospholipases and aspartyl proteinases, enzymes that play critical roles in tissue invasion and immune evasion, thereby enhancing the virulence of the biofilm [25, 26]. Additionally, another transcriptomic study of *C. auris* biofilms identified distinct pathogenic features, such as ferroptosis, a form of iron-dependent cell death, and the downregulation of DNA/RNA replication and repair mechanisms in host cells [5]. This downregulation contributes to the pathogen’s ability to impair host cell function and promote infection.

A notable characteristic of *C. auris* biofilms that enhance its pathogenesis is their ability to withstand environmental stresses. Unlike planktonic cells, *C. auris* biofilms exhibit increased resistance to oxidative stress, nutrient limitation, and pH changes, which are commonly encountered in the host environment [8]. Finally, the biofilm environment enahnce the survival of a heterogeneous population of cells, including dormant persisters that can resist harsh conditions [27]. These persister cells play a critical role in the recurrence of infections, as they can survive treatment and later repopulate the biofilm. The biofilm’s complex architecture and cellular diversity also contribute to its ability to evade the host immune response, making infections caused by *C. auris* biofilms particularly difficult to treat and control. The combination of these factors—upregulated pathogenic genes, enhanced stress resistance, and the presence of persister cells—makes *C. auris* biofilms a formidable challenge in clinical settings, leading to infections that are more severe, persistent, and resistant to conventional treatments.

### Persistence in healthcare settings and challenges in infection control

*C. auris* biofilms are particularly persistent in healthcare environments. They form on medical devices (e.g., catheters, prosthetic implants) and surfaces within the healthcare setting. The biofilm matrix protects the fungal cells from both environmental stresses and antimicrobial agents, allowing the biofilm to endure for extended periods even after cleaning attempts. Further, Standard disinfectants and cleaning agents are often ineffective against *C. auris* biofilms [28]. The extra cellular matrix of the biofilm acts as a barrier to disinfectants, allowing the biofilm to survive despite routine cleaning efforts. This necessitates the use of more aggressive cleaning protocols and specialized disinfectants to address biofilm contamination. Additionally, detecting *C. auris* biofilms can be challenging due to their ability to form on diverse surfaces and within complex environments. Specialized diagnostic techniques and enhanced surveillance are required to identify and manage biofilm-associated infections effectively. Unlike planktonic cells, controlling *C. auris* biofilm infections requires stringent infection control measures. This includes strict cleaning protocols, use of specialized disinfectants, and strict adherence to infection prevention practices. Outbreaks involving *C. auris* biofilms can be particularly difficult to control due to the pathogen’s persistence on medical devices and surfaces [29]. Coordinated efforts are required to manage outbreaks, including environmental decontamination, patient isolation, and enhanced infection control practices [29].

## *Candida auris* biofilm development, structure and composition

The biofilm formed by *Candida auris* is a complex, multi-layered structure that contributes significantly to its resistance to treatment and persistence in healthcare environments. Understanding the development, structure and composition of *C. auris* biofilms is essential for developing effective strategies to combat these infections.

The initial stage of biofilm formation involves the adhesion of *C. auris* cells to surfaces, which can be both biotic and abiotic [6]. This adhesion is mediated by specific adhesins and surface proteins, with glycosylphosphatidylinositol (GPI)-anchored cell wall genes such as *IFF4*,* CSA1*,* PGA26*, and *PGA52* being upregulated during this phase [21]. The importance of these genes is highlighted by the observation that an iff4Δ null mutant displayed decreased adhesion during the early stages of biofilm formation, as well as attenuated virulence [30]. Following initial adhesion, cells begin to proliferate and form clusters. These cell clusters are the foundational elements of the biofilm and contribute to its overall mass and structure. During proliferation phase, several genes are upregulated such as *RDC3*,* SNQ2*,* CDR1*,* and YHD3* [21]. These genes encoding efflux pumps that provide protection to the biofilm during biofilm formation. Additionally, genes responsible for producing components of the extracellular matrix, such as *KRE6* and *EXG* (which encode glucan-1,3-beta-glucosidase), are also upregulated, facilitating the biofilm’s structural development [21]. Over time, these clusters grow into a mature biofilm, which is characterized by a more complex and organized structure. Mature *Candida* biofilms often exhibit multiple layers of fungal cells interspersed with channels and voids that facilitate nutrient and waste exchange [31]. The extracellular matrix of the mature *C. auris* biofilm is rich in polysaccharides, such as beta-glucans and mannans [19]. These polysaccharides form a gel-like matrix that provides structural support to the biofilm and acts as a barrier to antimicrobial agents. *C. auris* biofilm matrix also contains various proteins that contribute to biofilm stability and cell-cell adhesion. These proteins can also interact with host tissues and immune components. A study by Paudyal and Vediyappan identified several proteins that are overexpressed in *C. auris* biofilms compared to planktonic cells, including Spe3p, Tdh3p, Sod2p, Ywp1p, and Mdh1p, further emphasizing the distinct composition and resistance of the biofilm [32]. As the biofilm matures, it continues to grow and expand, with ongoing cell proliferation and matrix production. The architecture of the biofilm becomes increasingly intricate, allowing it to persist for extended periods on surfaces. Cells within the biofilm can also disperse to colonize new surfaces, leading to the formation of additional biofilms, thereby perpetuating the infection cycle [33].

## Nanoparticles: types and mechanisms of action against *Candida auris* biofilms

Nanoparticles offer innovative approaches to tackling *Candida auris* biofilms, which are difficult to manage with conventional antifungal treatments. The unique properties of nanoparticles, such as their small size, large surface area, and ability to be functionalized with various chemical groups, enable them to disrupt biofilm formation and enhance antifungal activity in several ways. List of types of nanoparticles with antibiofilm activity are presented in Table 1.

**Table 1:** Strengths and limitations of various nanoparticle types for biofilm treatment

Nanoparticle type	Strengths	Weaknesses	Reference
Metallic (e.g., Silver, Zinc Oxide)	Strong antibiofilm activity, ROS generation, disrupts cell integrity	Potential cytotoxicity (e.g., silver), the tendency to agglomerate	[93]
Polymeric (e.g., Chitosan-based)	Targeted delivery, controlled release, can carry multiple drugs	Expensive to produce at scale	[94]
Lipid-based (e.g., Liposomes)	High biocompatibility, controlled release	Limited stability, potential for degradation, complex production and storage requirements	[74]
Nanoemulsions	Improves the pharmacokinetics, drug solubility and enables targeted drug delivery	Stability concerns, potential for phase separation, may require specific surfactants	[95]
Cyclodextrin-based	Increases solubility of encapsulated agents	Potential renal toxicity for some CDs, limited use in nasal administration	[96]
Nanofibers	High surface area, can carry bioactive agents	Production challenges with limited control over pore size	[97]

### Types of nanoparticles with antibiofilm activity

#### Nanoemulsions

Nanoemulsions are colloidal systems consisting of nanometer-sized droplets of one liquid dispersed within another immiscible liquid, stabilized by surfactants or emulsifiers [34]. These systems are particularly advantageous for drug delivery due to their unique properties and capabilities. The small droplet size of nanoemulsions, typically ranging from 20 to 500 nanometers, contributes to their stability and ability to penetrate biological barriers [35]. Nanoparticles with sizes specially below 500 nm are particularly effective in penetrating the ECM of *C. auris* biofilms, as this compact structure contains dense polysaccharides, proteins, and other components that block larger particles. Smaller nanoparticles can more easily weave through this network, reaching embedded fungal cells that are shielded by the biofilm’s matrix. Studies have shown that nanoparticles around 40 nm in size, such as certain nanoemulsions, significantly enhance the penetration of antifungal agents through *C. auris* biofilms by navigating these narrow pathways, ultimately leading to higher drug delivery efficiency [36]. Also, smaller nanoparticles, due to their high surface-area-to-volume ratio, have increased stability within biofilms and improved diffusion through the biofilm’s water channels [12, 37]. This allows for more uniform distribution of the drug within the biofilm structure. On the other hand, the surface charge of nanoparticles is crucial for biofilm interaction that consequently will result into better biofilm penetration. Positively charged nanoparticles have enhanced attraction to the negatively charged components of the *C. auris* biofilm matrix, such as polysaccharides and proteins [38]. This interaction allows them to adhere closely to the biofilm and infiltrate its structure more effectively. The high surface area-to-volume ratio of nanoemulsions enhances the interaction between the drug and its target, improving the bioavailability of encapsulated agents [39]. These systems are highly relevant in drug delivery, including for targeting biofilms [40]. For instance, a significant difference was observed in *C. auris* biofilm viability between amphotericin B loaded into nanoemulsion formulation (NEA) and free amphotericin B (AmB) for all concentrations tested. For AmB, the adhesion biofilm viability ranged from 93 to 100% at concentrations of 0.19, 0.09, 0.04, and 0.02 µg/mL, indicating that the biofilm was largely unaffected by the treatment at these concentrations. In contrast, the viability results for NEA at the same concentrations were markedly lower, at 17%, 47%, 49%, and 67%, respectively. These results clearly demonstrate a superior antibiofilm action for NEA compared to free AmB [36]. In another study, the antifungal activity of AmB and NEA were evaluated to inhibit the biofilm formation of four clinical isolates of *C. auris* from different clades. NEA exhibited significant inhibition potential compared to the free AmB against the (India, CLADE I—InP13) and VEN C6 (Venezuelan, CLADE IV) *C. auris* strains at concentrations of up to 0.07 µg/mL. Moreover, NEA was more effective than AmB in inhibiting mature biofilms across all the isolates from the four different clades [41].

Nanoemulsions can significantly improve the solubility and stability of hydrophobic and hydrophilic antimicrobial agents [42]. The nanoscale droplets in a nanoemulsion can encapsulate these agents, allowing them to be solubilized in an aqueous environment. This enhanced solubility ensures that higher concentrations of the drug can be delivered, which is particularly useful for drugs with poor water solubility [42]. The small droplet size of nanoemulsions allows for better penetration through the dense extracellular matrix of biofilms [40]. Nanoemulsions can navigate through this matrix more effectively, delivering their payload directly to the fungal cells within the biofilm. Nanoemulsions can be engineered to provide controlled and sustained release of antimicrobial agents. This means that the drug can be released gradually over time, maintaining therapeutic levels for an extended period [43]. This controlled release helps in achieving prolonged exposure of the biofilm to the drug, which can enhance the overall effectiveness of the treatment.

#### Metallic nanoparticles

Metallic nanoparticles have emerged as a promising solution for addressing the challenges posed by biofilms. The unique properties of metallic nanoparticles, such as their nanoscale size, large surface area, and high reactivity, enable them to effectively target and disrupt these resistant microbial communities [44, 45]. The ability of metallic nanoparticles to penetrate the extracellular matrix is a critical factor in their antibiofilm activity [46]. This matrix acts as a physical barrier that shields the microorganisms within from antimicrobial agents and the host immune response. Metallic nanoparticles, due to their small size, can infiltrate this matrix and reach the microbial cells embedded deep within the biofilm [47]. Once inside, these nanoparticles can destabilize the biofilm’s structure by interacting with the matrix components, weakening its protective barrier and making the biofilm more susceptible to treatment [48].

In addition to physically disrupting the biofilm structure, metallic nanoparticles generate reactive oxygen species (ROS), which play a key role in their antimicrobial action [49]. These ROS induce oxidative stress within the biofilm, leading to damage of vital cellular components such as lipids, proteins, and nucleic acids [50]. The oxidative damage not only affects the microorganisms but also compromises the integrity of the extracellular matrix, further enhancing the vulnerability of the biofilm to antimicrobial agents. This dual action—disruption of the matrix and oxidative damage to microbial cells—makes metallic nanoparticles particularly effective against biofilms. Moreover, metallic nanoparticles can interfere with the quorum sensing mechanisms that bacteria and fungi use to regulate biofilm formation and maintenance [51]. Quorum sensing is a communication process that allows microorganisms to coordinate their behavior, including the production of virulence factors and the development of biofilms [52]. By disrupting these signaling pathways, metallic nanoparticles can prevent the initial formation of biofilms or destabilize existing ones, thereby reducing the biofilm’s ability to persist and resist treatment. Furthermore, the potential of metallic nanoparticles extends beyond merely killing the microorganisms within a biofilm. Their application can also prevent the reformation of biofilms after treatment, offering a long-term solution to biofilm-related problems. The versatility of metallic nanoparticles allows them to be incorporated into coatings, textiles, and other materials, providing continuous protection against biofilm formation in a variety of environments [38]. This is particularly important in medical settings, where biofilm formation on devices such as catheters and implants can lead to chronic infections and complicate patient care. By preventing biofilm formation, metallic nanoparticles help reduce the risk of recurrent infections and improve the efficacy of medical treatments [53].

#### Polymeric nanoparticles

Polymeric nanoparticles are an emerging solution for combating biofilms, one of the key advantages of polymeric nanoparticles is their ability to enhance drug delivery [54–56]. They can be engineered to deliver antimicrobial agents directly to the biofilm site, which increases the local concentration of the drug and helps overcome the biofilm’s protective barrier [57]. Moreover, these nanoparticles can be designed for controlled and sustained release of antimicrobial agents, ensuring prolonged exposure to the biofilm and improving the chances of disrupting it [58].

Another important feature of polymeric nanoparticles is their ability to penetrate the biofilm matrix [59]. Their small size allows them to infiltrate the dense extracellular matrix, and surface modifications, such as coating with hydrophilic polymers like polyethylene glycol (PEG), can enhance this penetration [60]. In some cases, polymeric nanoparticles are designed to carry enzymes or toxin that degrade the biofilm matrix, making it more susceptible to antimicrobial agents [60, 61]. Some polymeric nanoparticles also possess intrinsic antimicrobial properties. For instance, cationic polymers like chitosan can disrupt microbial cell membranes due to their positive charge [62, 63]. By incorporating these polymers into nanoparticles, an additional mechanism of action against biofilms is achieved. Polymeric nanoparticles can also be loaded with multiple antimicrobial agents or combined with other therapeutic strategies, such as quorum sensing inhibitors, providing a multifaceted approach to biofilm eradication. For instance, in a study focusing on *Pseudomonas aeruginosa*, researchers developed hydrophobic nanoparticles by grafting 11-carbon and three-carbon alkyl chains to a chitosan polymer, aiming to enhance the delivery of carvacrol for its antibacterial and antibiofilm properties. The study demonstrated that nanoparticles containing the 11-carbon chain modified chitosan exhibited superior penetration through the biofilm compared to those made from unmodified chitosan. By assessing the interaction of these nanoparticles with a 50:50 w/w phospholipid mixture at the air–water interface, the researchers concluded that the viscoelastic and fluidity properties of the nanoparticles were enhanced. This led to a notable reduction in viable *P. aeruginosa* cells in biofilms. Furthermore, these nanoparticles effectively reduced quorum sensing in *Chromobacterium violaceum* [64]. Translating this approach to *C. auris*, the incorporation of hydrophobic nanoparticles that carry quorum-sensing inhibitors could enhance the treatment of *C. auris* biofilms. By modifying the surface properties of chitosan nanoparticles, we can improve their interaction with the fungal cell membranes, facilitating deeper penetration into preformed biofilms. This strategy could significantly lower the virulence and pathogenicity of *C. auris*, similar to the observed outcomes in *P. aeruginosa*, by disrupting quorum sensing and reducing biofilm resilience.

Further, the polymeric nanoparticles can also help overcome drug resistance [65]. By encapsulating antibiotics or antifungal agents within polymeric nanoparticles, the drug is protected from enzymatic degradation by biofilm-forming microorganisms, thereby maintaining its efficacy [66]. Additionally, multifunctional nanoparticles can be designed to target resistance mechanisms, such as efflux pump inhibitors or agents that interfere with resistance-conferring genes, helping to restore the effectiveness of conventional drugs [67, 68]. Targeting biofilms specifically is another strength of polymeric nanoparticles. They can be functionalized with ligands that specifically target components of the biofilm or bacterial/fungal surface structures, which reduces off-target effects and increases the local concentration of the therapeutic agent at the biofilm site [69]. For instance, specific ligands such as antibodies (e.g., anti-adhesion antibodies) [70], or antifungal peptides [71], can be utilized for nanoparticle functionalization. By attaching these ligands to nanoparticles, we can enhance their binding to the biofilm matrix and the microbial surface. Antibodies can provide high specificity to target surface structures, such as adhesins involved in biofilm formation, while peptides can disrupt cell membrane integrity or inhibit biofilm development. Additionally, pH-sensitive polymeric nanoparticles can be designed to release their payload in response to the acidic conditions within biofilms, ensuring targeted delivery [72].

#### Lipid-based nanoparticles

Lipid-based nanoparticles are gaining attention as a promising approach to combat biofilms [66]. They typically consist of a lipid core that encapsulates hydrophobic drugs, surrounded by a hydrophilic shell [73]. This amphiphilic structure enables them to interact effectively with both the biofilm matrix and the fungal cells within it. The most common types of lipid-based nanoparticles include solid lipid nanoparticles, nanostructured lipid carriers, liposomes, and lipid-polymer hybrid nanoparticles, each offering unique advantages in terms of stability, drug loading capacity, and controlled release.

One of the key advantages of lipid-based nanoparticles, such as liposomes and solid lipid nanoparticles, is their biocompatibility and ability to encapsulate both hydrophobic and hydrophilic drugs [74]. This versatility enables the delivery of a wide range of antimicrobial agents directly to the biofilm site [75–77]. By encapsulating these agents, lipid-based nanoparticles can protect the drug from degradation, enhance its stability, and improve its penetration into the biofilm matrix. Lipid-based nanoparticles are particularly effective in penetrating the biofilm’s extracellular matrix [78]. Their small size and lipid composition allow them to interact with the biofilm structure, facilitating deeper penetration of the antimicrobial agents they carry [79]. This penetration is crucial for reaching and eradicating the microbial cells embedded within the biofilm, which are often shielded from conventional treatments. Additionally, lipid-based nanoparticles can be designed to release their payload in a controlled and sustained manner [74]. This ensures prolonged exposure of the biofilm to the antimicrobial agent, increasing the likelihood of disrupting the biofilm structure and killing the embedded microorganisms. Some lipid-based nanoparticles are also engineered to be responsive to environmental triggers, such as pH or temperature changes, which are often characteristic of biofilm environments [80, 81]. This trigger-responsive release further enhances the targeted delivery of antimicrobial agents. Lipid based nanoparticles can also be designed to co-deliver multiple therapeutic agents, including antifungal drugs and antimicrobial adjuvants that can enhance the efficacy of the antifungal drug. This multi-faceted approach can help overcome the inherent resistance mechanisms of biofilms, such as the restricted penetration of drugs and the presence of persister cells that are tolerant to conventional antifungal treatments [82].

Similarly to polymeric nanoparticles, lipid-based nanoparticles also offer the potential for biofilm-specific targeting. By functionalizing their surface with ligands that bind specifically to biofilm components or microbial surface structures, lipid-based nanoparticles can achieve targeted delivery, reducing off-target effects and increasing the effectiveness of the encapsulated antimicrobial agent [82].

#### Cyclodextrin nanoparticles

Cyclodextrin-based nanomaterials have emerged as a promising approach in the treatment of biofilm-associated infections due to their unique structural and chemical properties [57]. Cyclodextrins (CDs) are cyclic oligosaccharides composed of glucose units that form a hydrophilic exterior and a hydrophobic cavity, allowing them to encapsulate a variety of guest molecules [83]. This capability makes CDs particularly effective as carriers for delivering biofilm-disrupting agents and antimicrobial compounds directly to the site of infection.

One of the key advantages of cyclodextrin-based nanomaterials is their ability to enhance the solubility and stability of poorly water-soluble antimicrobial agents, which can be crucial for effective delivery to the biofilm [84, 85]. Many antimicrobial drugs, particularly hydrophobic ones, struggle to penetrate the dense biofilm matrix. By encapsulating these drugs within the hydrophobic cavity of CDs, their solubility is improved, and they can be delivered more effectively to the target site [86].

Another significant benefit of cyclodextrin-based nanomaterials is their capacity for targeted delivery and controlled release of antimicrobial agents [87]. CDs can form inclusion complexes with hydrophobic drugs, enhancing their penetration into biofilms and enabling controlled release of the encapsulated drug. This controlled release ensures sustained therapeutic levels over an extended period, which is essential for completely eradicating biofilms. Cyclodextrin-based nanomaterials also hold the potential to reduce the development of drug resistance in biofilm-associated infections [84]. By delivering drugs in a targeted and controlled manner, they minimize the exposure of non-target bacteria to subtherapeutic drug concentrations, which is a common driver of resistance. Moreover, the biocompatibility and safety of CDs, which are generally recognized as safe (GRAS) by regulatory agencies, make them an attractive option for clinical applications. Their favorable safety profile reduces the risk of adverse reactions, making them suitable for a wide range of patients [88].

#### Nanofibers

Nanofibrous materials are ultra-fine fibers with diameters typically in the nanometer range, created using techniques like electrospinning [89]. These materials have a high surface area-to-volume ratio, tunable porosity, and can be functionalized with various bioactive agents, making them an effective tool against biofilm formation [90]. Nanofibrous materials act as anti-biofilm agents in several ways. Their high surface area allows for the effective delivery and sustained release of antifungal agents, ensuring prolonged contact with the fungal cells [90]. Additionally, the surface of nanofibers can be modified with antifungal agents or coated with substances like silver nanoparticles, which possess inherent antifungal properties [91]. These modifications can disrupt the biofilm structure by interfering with the adhesion of fungal cells, a critical step in biofilm formation. The mechanical properties of nanofibrous materials also contribute to their anti-biofilm activity, as they can physically interact with and disrupt the biofilm matrix, preventing the establishment and maturation of biofilms [90]. Moreover, nanofibers can be designed to mimic the extracellular matrix of host tissues, thereby acting as a competitive surface that hinders microbial cells from adhering to and colonizing actual tissues or medical devices [92]. This competitive adhesion prevents the initial establishment of the biofilm, making it easier to control or eradicate the fungal cells before they form a resilient biofilm.

### Mechanisms of action against *Candida auris* biofilms

**Fig. 1:**
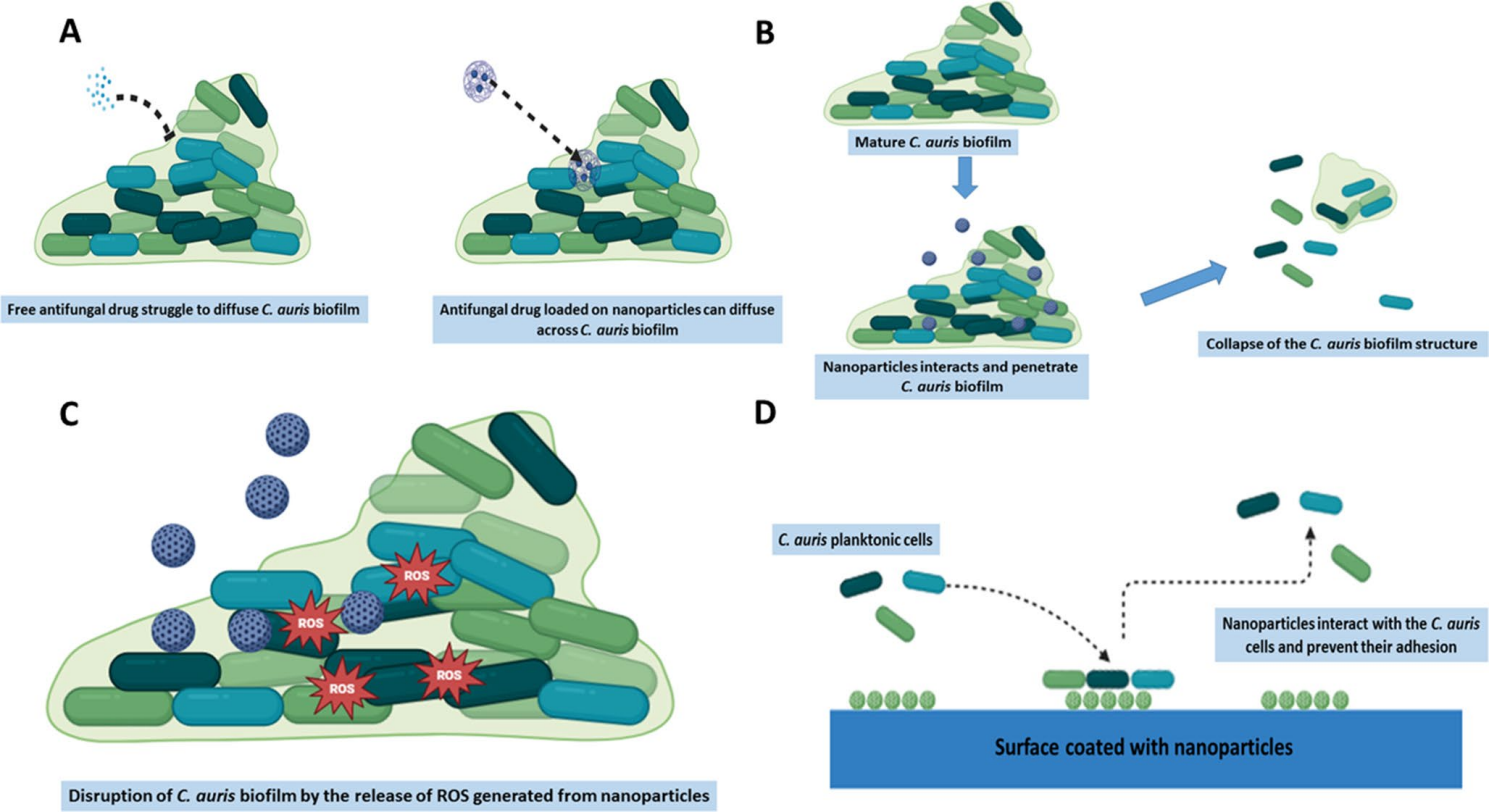
Mechanism of nanoparticles against *C. auris* biofilm. (**A**) Nanoparticles enhance antifungal penetration, (**B**) Nanoparticles disrupt *C. auris* biofilm structure by direct interacting with the biofilm matrix, (**C**) Nanoparticles release intracellular ROS, (**D**) Nanoparticles prevent *C. auris* adhesion and consequently biofilm formation

Nanoparticles have shown significant potential in combating *C. auris* biofilms through various mechanisms of action as indicated in Table 2 and Fig. 1. These mechanisms are tailored to overcome the protective barriers of the biofilm and directly target the fungal cells within, thereby enhancing the efficacy of antifungal treatments.

**Table 2:** Nanoparticles employed to combat *Candida auris* biofilm

Type of nanoparticle	Composition	Particle size	Mechanism of action	Reference
Nanoemulsions	10% of oil phase consist of sunflower oil and cholesterol (5:1), 80% of PBS and 10% of surfactant (Brij^®^ 58/SPA (2:1)	40 nm	Deliver amphotericin b through *C. auris* biofilm matrix	[36, 41]
Liposomes	L-α-phosphatidylcholine and cholesterol (70/30 mol/mol)	177.2 ± 11.4 nm	Deliver essential oil through *C. auris* biofilm matrix and generation of ROS	[98]
Metallic nanoparticles	Silver coated with polyvinylpyrrolidone	6.18 ± 5 nm	Disruption of *C. auris* biofilm cellular integrity through the release of silver ions	[99]
Metallic nanoparticles	Silver coated with polyvinylpyrrolidone	15–20 nm	Disruption of *C. auris* biofilm cellular integrity through the release of silver ions	[11]
Metallic nanoparticles	Silver	1 to 3 nm	Disruption of *C. auris* biofilm cellular integrity through the release of silver ions. Prevent biofilm formation by inhibiting the fungus surface adhesion	[38]
Metallic nanoparticles	Bismuth	8 nm	Disruption *C. auris* cell morphology and the overall architecture of the biofilms	[100]
Nanofibrous membrane	polylactic acid-hypocrellin A	423–699 nm	Release of endogenous ROS levels	[101]
Metallic nanoparticles	Zinc oxide	50 ng	Interfere with cell adhesion by downregulate *C. auris* adhesive gene *ALS5*	[10]
Metallic nanoparticles	Silver	Less than 100 nm	Prevent *C. auris* biofilm formation by inhibiting the adhesion of cells to the coated surface.	[102]

#### Deliver antimicrobial drugs across the biofilm matrix

Nanoparticles can be engineered to encapsulate and deliver antifungal drugs directly to biofilm-embedded cells as illustrated in Fig. (1 A). The enhanced penetration of antimicrobial drugs through the *C. auris* biofilm matrix is a critical aspect of addressing biofilm-associated infections. Nanoparticles are particularly effective in penetrating the biofilm matrix due to their small size, surface properties, and ability to be engineered for specific interactions with the biofilm components. Unlike conventional drugs, which may struggle to diffuse through the dense biofilm matrix, nanoparticles can navigate through this protective barrier and deliver their payload directly to the fungal cells embedded within the biofilm. Several factors contribute to the enhanced penetration of nanoparticles into the biofilm matrix, including size, shape, particle charge, and the ability to encapsulate antimicrobial drugs. While several reports highlighted the application of nanoparticles to enhance penetration of antimicrobial drugs through biofilm [103–106], only two studies were found addressing *C. auris* biofilm. This study demonstrated that nanoemulsions could be used to deliver antifungal drugs across *C. auris* biofilms [36]. Specifically, oval-shaped nanoemulsions with a size of 40 nm were effective in enhancing the activity of amphotericin B against *C. auris* biofilms. The nanoemulsion formulation included cholesterol, which facilitated interaction with ergosterol in the *C. auris* cell membrane, improving drug penetration and efficacy. Additionally, the nanoemulsion extended the release of amphotericin B for 12 h. Another study highlighted the potential of liposome to act as drug carrier across *C. auris* biofilm [98]. In this study, liposome-encapsulated essential oil from *Lavandula angustifolia.* Showed promising activity to eradicate *C. auris* biofilm. Liposomes are small, spherical vesicles composed of one or more lipid bilayers, which can encapsulate drugs or other therapeutic agents within their aqueous core or lipid layers. They are often used as drug delivery systems due to their ability to carry a wide range of therapeutic compounds, including both hydrophilic and hydrophobic substances [107]. Liposomes can penetrate biofilms and deliver their payloads effectively as the lipid bilayer of liposomes is similar in structure to the cell membranes of microbes, including those within biofilms. These promising findings underscore the potential of nanoemulsions and liposomes to deliver antimicrobial drug within *C. auris* biofilm; however, more research is needed to further develop and refine other types of nanoparticle-based approaches to deliver drugs across *C. auris* biofilms.

#### Disruption of cellular integrity

The disruption of cellular integrity by nanoparticles represents a promising approach in combating resilient microbial biofilms, including those formed by *C. auris*. Nanoparticles possess unique physicochemical properties, such as high surface area-to-volume ratio, which enables them to interact closely with microbial cells and biofilm structures (Fig. 1B). This interaction can induce oxidative stress, membrane destabilization, and disruption of vital cellular processes, ultimately leading to the collapse of the biofilm structure and the death of the fungal cells. Silver nanoparticles (AgNPs) are well-known for their potent antimicrobial properties, which stem from their unique physicochemical characteristics, including a high surface area-to-volume ratio and the ability to release silver ions [11, 38, 99, 108]. These properties enable AgNPs to interact closely with microbial cells and biofilm structures. They have a strong affinity for the negatively charged components of microbial cell membranes, such as phospholipids and proteins. Upon interaction, AgNPs can insert themselves into the cell membrane, causing structural disruptions. In the context of *C. auris*, this interaction can lead to increased membrane permeability, leakage of intracellular contents, and ultimately, cell lysis. Vazquez-Munoz study have highlighted the potent antibiofilm activity of AgNPs across *C. auris* strains from different clades, where they disrupted biofilm formation and altered the shape and size of individual cells, with a noticeable reduction in cell clustering [99]. Similar findings were reported by AlJindan and AlEraky, who observed the effectiveness of AgNPs in combating *C. auris* biofilms in clinical isolates [11]. In another study, the AgNPs with diameter of 1 to 3 nm were effective to eradicate preformed *C. auris* biofilm at IC_50_ of 0.48 ppm [38]. In comparison, bismuth nanoparticles (BiNPs) have also shown promising results against *C. auris* biofilm integrity. One of the key advantages of BiNPs is their relatively low toxicity compared to other heavy metal-based nanoparticles, making them a safer alternative for clinical use [109]. BiNPs exhibited antibiofilm activity against *C. auris* strains from four different clades with IC_50_ ranging from 5.1 to 113.1 µg/ml. BiNPs exhibited strong antibiofilm activity against *C. auris* strains from four different clades, with IC_50_ values ranging from 5.1 to 113.1 µg/ml. At the structural level, BiNPs disrupted *C. auris* cell morphology and the overall architecture of the biofilms, further underscoring their potential as effective tools in combating this challenging pathogen [100]. Further research and optimization of these nanoparticles could pave the way for their broader application in clinical settings.

#### Generation of reactive oxygen species

The generation of reactive oxygen species (ROS) has emerged as a powerful tool in the fight against *C. auris* biofilms (Fig. 1C) [110]. ROS, including hydrogen peroxide, superoxide anions, and hydroxyl radicals, are highly reactive molecules that can induce significant oxidative stress within microbial cells. This oxidative stress can lead to substantial damage to various cellular components, including lipids, proteins, and DNA, thereby compromising the integrity of the cell membrane and the biofilm structure. Disruption of *C. auris* biofilm by the generation of ROS was observed by encapsulating essential oil into liposome [98]. Increase in the intracellular ROS and apoptotic pathway in *C. auris* biofilm was concentration dependent following treatment with liposome-encapsulated essential oil. The study highlighted the potential role of the transcription factor *HOG1* during the oxidative stress induced by the intracellular release of ROS. Another study demonstrated that a biodegradable nanofibrous membrane made of polylactic acid-hypocrellin A (PLA-HA), when used in combination with antimicrobial photodynamic therapy, can eliminate *C. auris* biofilm formation. This is achieved by increasing endogenous ROS levels, which result in the dysfunction of mitochondria together with the release of cytochrome C and the activation of metacaspase [101].

#### Interference with cell adhesion

Nanoparticles can interfere with the initial adhesion of *C. auris* cells to surfaces, preventing biofilm formation (Fig. 1D). Nanoparticles have shown significant promise in preventing the initial adhesion of *C. auris* cells to surfaces, thereby inhibiting biofilm formation [10, 38, 102, 108]. The initial adhesion of microbial cells to surfaces is a critical first step in biofilm development, and disrupting this process can effectively prevent the establishment of a biofilm. Nanoparticles can interfere with this initial adhesion through several mechanisms. Firstly, the high surface area and reactivity of nanoparticles enable them to interact closely with microbial cells and surfaces. By coating or embedding surfaces with nanoparticles, their presence can alter the physical and chemical properties of the surface, making it less conducive for microbial adhesion. This modification can hinder the ability of *C. auris* cells to adhere and initiate biofilm formation. Additionally, nanoparticles can directly interact with the surface structures of *C. auris*, such as the cell wall and adhesins, which are crucial for the initial adhesion process [10]. Certain nanoparticles can bind to these surface molecules, blocking the interaction sites and preventing the cells from anchoring to the surface. This direct interference reduces the likelihood of successful adhesion and subsequent biofilm development. For instance, ZnO NPs can downregulate *C. auris* adhesive gene *ALS5* 7.2 fold [10]. The *ALS* (Agglutinin-Like Sequence) gene family plays a significant role in the biofilm formation of *Candida* species. The *ALS* genes encode a family of cell surface glycoproteins that contribute to the adhesion of *Candida* cells to host tissues and abiotic surfaces, which is a critical step in biofilm development [111, 112]. The study of Lara et al., showed that silicone elastomers functionalized with AgNPs can prevent the *C. auris* biofilm formation by inhibiting the pathogen surface adhesion [38]. Similar observation was indicated when medical dressing was coated with AgNPs [38]. In another study, polycaprolactone blended with calcium phosphates contains silver prevented *C. auris* biofilm formation by inhibiting the initial cell adhesion Further, AgNPs cluster coatings were developed on copper surfaces prevented the adhesion of *C. auris* and consequently the biofilm formation [102].

## Translational potential of nanoparticle-based strategies against *C. auris* biofilms

**Fig. 2:**
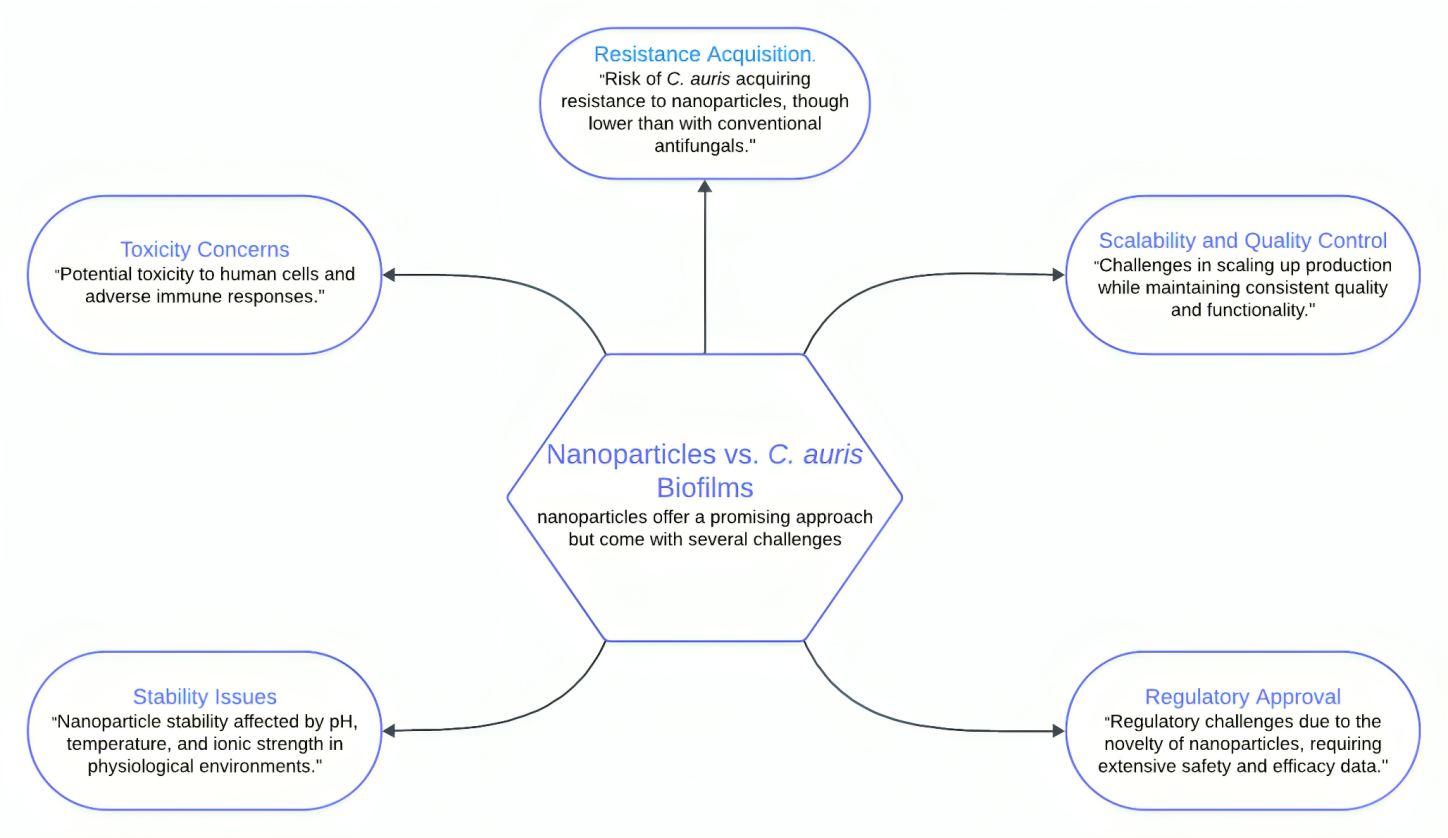
Challenges and considerations in using nanoparticles against *Candida auris* biofilms

Nanoparticle-based strategies for combating *C. auris* biofilms hold significant promise, but their successful clinical translation will depend on overcoming several practical challenges and demonstrating clear advantages over existing therapies. From a translational perspective, one of the primary considerations is the scalability and reproducibility of nanoparticle synthesis. Unlike small-molecule drugs, nanoparticles are complex systems whose therapeutic efficacy is highly dependent on parameters such as size, shape, surface charge, and functionalization. Ensuring that these properties remain consistent across large-scale production is crucial for maintaining therapeutic efficacy and minimizing batch-to-batch variability. This consistency is not only important for clinical application but is also a key regulatory requirement, as variability can affect pharmacokinetics and therapeutic outcomes. Additionally, applying nanoparticles against *C. auris* biofilms involves several challenges, including the organism’s potential to acquire resistance when exposed to antifungal drugs (Fig. 2). *C. auris* is known for rapidly developing resistance to multiple antifungal classes, which complicates treatment [113]. However, unlike conventional antifungal drugs, nanoparticles are less likely to induce resistance because they often act non-specifically or possess multiple mechanisms of action [45]. This multi-targeted approach reduces the chance of the pathogen developing resistance. Nonetheless, it is essential to conduct thorough analyses to ensure that *C. auris* does not acquire resistance to nanoparticles upon repeated exposure, particularly as nanoparticles become more widely used in clinical settings. Another challenge is the potential for nanoparticle toxicity. While nanoparticles can be effective against biofilms, they must be carefully designed to avoid harming human cells or causing adverse immune responses [114]. The stability of nanoparticles in physiological environments is also a concern, as factors like pH, temperature, and ionic strength can affect their efficacy [115]. Scaling up the production of nanoparticles presents a significant challenge, particularly when it comes to maintaining consistent quality and functionality across larger batches. As production scales up, variations in the synthesis process can lead to inconsistencies in size, shape, and surface characteristics of the nanoparticles, which can directly affect their efficacy and safety. These variations could compromise the nanoparticles’ ability to penetrate biofilms, and interact with *C. auris*. Additionally, ensuring that the nanoparticles remain stable and effective under industrial production conditions is essential to meet regulatory standards and achieve successful clinical outcomes. Therefore, strict quality control measures and process optimization are critical to overcoming these hurdles and ensuring that scaled-up nanoparticle production meets the necessary standards for widespread medical use [116]. Regulatory approval poses a challenge for the use of nanoparticles in medical applications, as they are relatively new to this field. Their long-term safety and efficacy must be thoroughly evaluated before they can be approved for treating *C. auris* biofilms. This rigorous evaluation process ensures that the nanoparticles meet al.l necessary safety standards and demonstrate effectiveness in clinical settings, which can be time-consuming and complex. The novelty of nanoparticles means that regulatory agencies require extensive data to assess their potential risks and benefits, adding to the difficulty of gaining approval for their use in medical treatments [117]. The clinical implementation of nanoparticle-based therapies also requires considerations of cost-effectiveness and patient compliance. Ultimately, the successful translation of nanoparticle-based antifungal therapies from bench to bedside will require a multidisciplinary effort, involving collaboration among chemists, microbiologists, clinicians, and regulatory experts. The ongoing advancements in nanoparticle engineering, coupled with a deeper understanding of *C. auris* pathogenesis and biofilm biology, will be instrumental in overcoming these translational hurdles and realizing the full therapeutic potential of these innovative nanotechnologies in clinical settings.

## Limitations and gaps in current research

While nanoparticle-based strategies show significant promise in addressing *C. auris* biofilms, several limitations and gaps in the current research must be addressed to optimize these approaches for clinical use. One major gap is the lack of standardized protocols for evaluating the efficacy of nanoparticles against *C. auris* biofilms in vitro and in vivo. Most studies utilize different nanoparticle formulations, testing conditions, and biofilm models, making it challenging to compare results across studies and draw definitive conclusions about the most effective strategies. Additionally, the biofilm matrices formed by *C. auris* vary significantly depending on the environmental conditions and clinical setting, yet current research does not fully account for these variations [2]. This variability could affect the penetration and activity of nanoparticles, underscoring the need for standardized biofilm models that mimic clinical scenarios more accurately. Another limitation is the limited understanding of the long-term safety and stability of nanoparticle formulations under physiological conditions. While many studies demonstrate promising antifungal efficacy, few investigate the potential cytotoxicity, immunogenicity, or long-term biodistribution of these nanoparticles in animal models. This is a critical gap, as nanoparticles that show high antifungal activity in vitro may have adverse effects in vivo, limiting their clinical applicability [118]. Moreover, there is a need to explore the interaction of nanoparticles with complex microbial communities and host immune responses, as biofilms in clinical settings are often polymicrobial and exist within a host environment that can influence their behavior and susceptibility [119]. Additionally, while nanoparticles are often proposed as solutions to drug resistance, few studies have explored whether *C. auris* can develop resistance to these nanomaterials over time [10]. Although nanoparticles tend to act through multiple mechanisms, the potential for resistance development cannot be entirely ruled out, particularly with repeated or subtherapeutic exposures. Research is needed to investigate whether resistance to nanoparticle-based therapies could emerge, and if so, to identify strategies to mitigate this risk. Lastly, the translation of laboratory findings into clinically viable treatments remains a significant gap. Most nanoparticle-based therapies are still in the early stages of research, with limited data on their performance in animal models or clinical trials. There is a pressing need to advance these studies to preclinical and clinical testing phases to establish their real-world efficacy and safety. Furthermore, addressing the challenges of large-scale production, formulation stability, and cost-effectiveness is essential for bringing these innovative therapies to market. Without addressing these gaps, the clinical utility of nanoparticle-based treatments against *C. auris* biofilms will remain uncertain.

## Future prospective

**Fig. 3:**
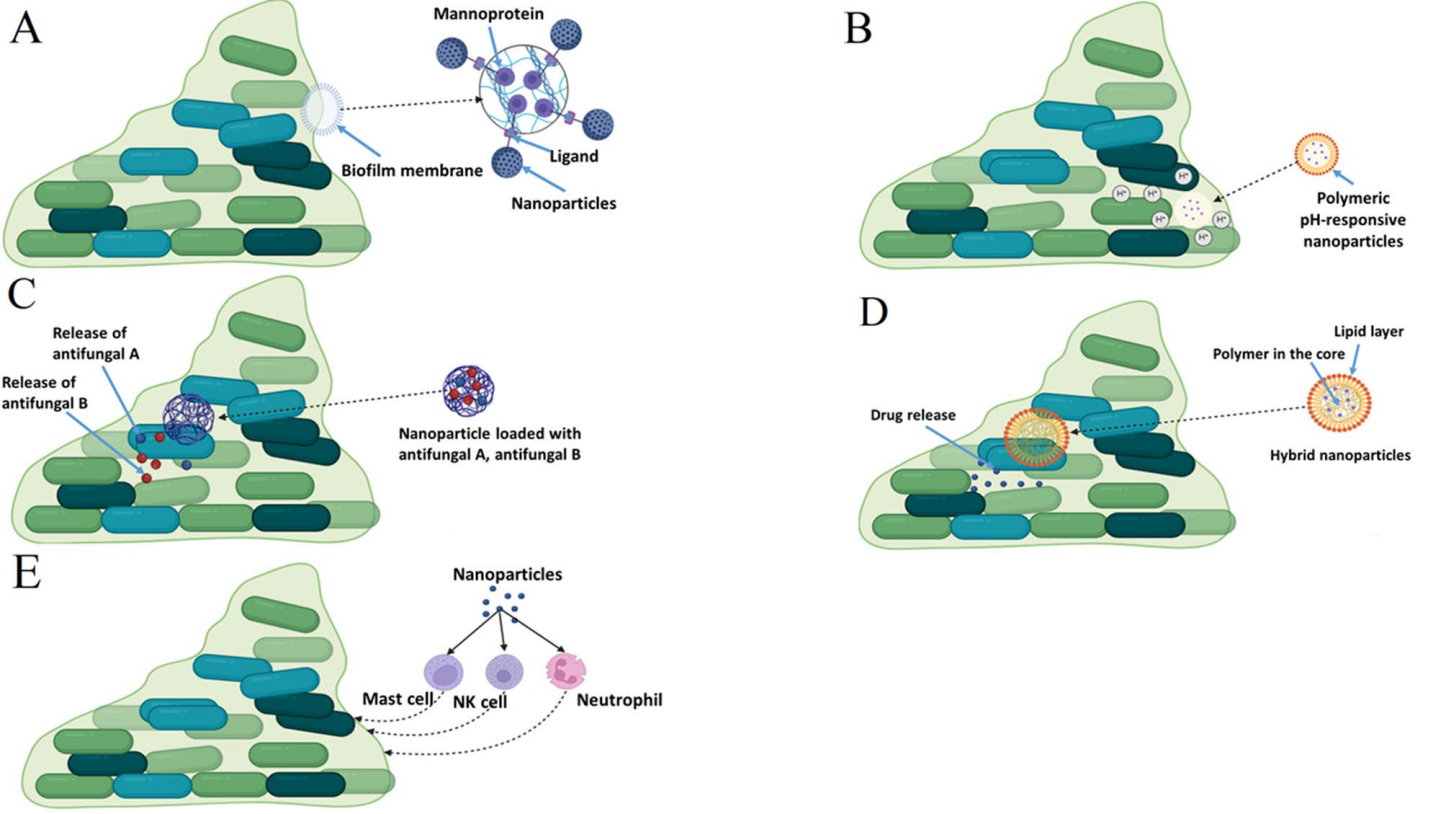
Future prospective for the nanoparticles application against *C. auris* biofilm. (**A**) nanoparticles functionalizing them with ligand target mannoprotein, (**B**) pH-responsive nanoparticles that release antifungal drug in the acidic environment of the *C. auris* biofilm, (**C**) nanoparticles that co-deliver multiple antifungal drugs, (**D**) lipid-polymer hybrid nanoparticles, (**E**) nanoparticles modulate immune activity against *C. auris* biofilm

As research into nanoparticles for treating *C. auris* biofilms advances, several promising directions and innovations are emerging, aiming to enhance the efficacy, safety, and practicality of nanoparticle-based therapies. Advanced nanoparticle design is one key area of progress. Developing targeted nanoparticles with specific targeting capabilities can improve their effectiveness against *C. auris* biofilms by functionalizing them with ligands or antibodies that recognize and bind to biofilm-specific components, thus enhancing selective targeting and reducing off-target effects. For instance, one promising ligand could be a peptide or antibody that binds to the polysaccharides or proteins present in the *C. auris* biofilm matrix, such as β-glucans or mannoproteins (Fig. 3A). Similarly, incorporating responsive elements into nanoparticles can create “smart” systems that release therapeutic agents in response to environmental triggers, such as pH changes or specific enzymes found in biofilm matrices (Fig. 3B). This approach can optimize drug delivery and minimize toxicity to healthy tissues. For instance, nanoparticles can be designed with polymeric coatings made from pH-responsive materials like poly (ethylene glycol)-b-poly (aspartic acid) or poly (acrylic acid). These materials undergo conformational changes in response to pH fluctuations. In the context of *Candida* biofilms, which often exhibit an acidic microenvironment compared to healthy tissue [120], such pH-sensitive nanoparticles can be engineered to remain stable at neutral pH but release their therapeutic agents when they encounter the acidic conditions of the biofilm [121]. This allows for targeted drug delivery directly within the biofilm environment, optimizing the release of antifungal agents precisely where they are needed and minimizing potential toxicity to surrounding healthy tissues. In addition, combination therapies are becoming increasingly important. Combining nanoparticles with conventional antifungal agents can enhance treatment efficacy, as nanoparticles can deliver antifungal drugs more effectively, improve drug penetration into biofilms, and potentially reduce the required dosage of antifungals. However, more research is required to address the loading of antifungal agents into nanoparticles to enhance their penetration across *C. auris* biofilms. This topic is crucial and not sufficiently discussed in the literature compared to bacterial biofilms. Furthermore, developing nanoparticles capable of co-delivering multiple drugs or therapeutic agents can target various aspects of biofilm formation and resistance simultaneously, addressing the complexity of biofilm infections more effectively than single-agent treatments (Fig. 3C).

Creating hybrid nanoparticles that combine different materials or functionalities can offer enhanced properties and performance. For example, metal nanoparticles composed of multiple elements, such as gold-silver or gold-copper nanoparticles, can be fabricated to exploit the unique optical and electronic properties of each metal, resulting in synergistic effects [45]. These hybrid metal nanoparticles can offer improved catalytic activity, enhanced imaging capabilities, or better antimicrobial properties compared to single-metal nanoparticles. Similarly, lipid-polymer hybrid nanoparticles represent another innovative approach (Fig. 3D). These nanoparticles combine the advantages of lipid and polymer components to achieve improved performance [74]. For instance, lipid layers can provide stability and controlled release of therapeutic agents, while polymer cores can enhance the mechanical strength and loading capacity of the nanoparticles. This hybrid design allows for better drug delivery efficiency, targeted release, and reduced toxicity, making them suitable for various biomedical applications, including targeting biofilms like those formed by *C. auris*.

Finally, integrative approaches are being considered. Exploring how nanoparticles interact with the host immune system could lead to innovative strategies that enhance immune responses against *C. auris* biofilms [122]. By designing nanoparticles that modulate immune activity, researchers aim to create systems that can either enhance the host’s natural immune response or specifically target immune cells to improve their ability to combat fungal infections (Fig. 3E). For example, nanoparticles can be engineered to deliver immune-boosting agents, such as cytokines or immunomodulatory drugs, directly to sites of infection. This targeted delivery can amplify the immune system’s response to *C. auris* biofilms, potentially increasing the effectiveness of the body’s defense mechanisms. Additionally, nanoparticles could be designed to present antigens or adjuvants that stimulate a stronger and more specific immune response against the biofilm-associated pathogens [123].

These strategies could help overcome the limitations of conventional treatments and offer more effective solutions for managing complex biofilm infections. By focusing on these integrative approaches, researchers hope to improve therapeutic outcomes and develop new treatment paradigms for *C. auris* biofilm-related infections.

## Data Availability

Not applicable.
